# A Quantitative Method to Monitor Reactive Oxygen Species Production by Electron Paramagnetic Resonance in Physiological and Pathological Conditions

**DOI:** 10.1155/2014/306179

**Published:** 2014-10-12

**Authors:** Simona Mrakic-Sposta, Maristella Gussoni, Michela Montorsi, Simone Porcelli, Alessandra Vezzoli

**Affiliations:** ^1^Istituto di Bioimmagini e di Fisiologia Molecolare, Consiglio Nazionale delle Ricerche, Via Fratelli Cervi 93, 20090 Segrate, Italy; ^2^Dipartimento di Fisiopatologia Medico-Chirurgica e dei Trapianti, Università di Milano, Via Fratelli Cervi 93, 20090 Segrate, Italy; ^3^Istituto per lo Studio delle Macromolecole, Consiglio Nazionale delle Ricerche, Via Bassini 15, 20133 Milano, Italy; ^4^Università Telematica S. Raffaele Roma, Via F. Daverio 7, 20122 Milano, Italy

## Abstract

The growing interest in the role of Reactive Oxygen Species (ROS) and in the assessment of oxidative stress in health and disease clashes with the lack of consensus on reliable quantitative noninvasive methods applicable. The study aimed at demonstrating that a recently developed Electron Paramagnetic Resonance microinvasive method provides direct evidence of the “instantaneous” presence of ROS returning absolute concentration levels that correlate with “a posteriori” assays of ROS-induced damage by means of biomarkers. The reliability of the choice to measure ROS production rate in human capillary blood rather than in plasma was tested (step I). A significant (*P* < 0.01) linear relationship between EPR data collected on capillary blood versus venous blood (*R* ^2^ = 0.95), plasma (*R* ^2^ = 0.82), and erythrocytes (*R* ^2^ = 0.73) was found. Then (step II) ROS production changes of various subjects' categories, young versus old and healthy versus pathological at rest condition, were found significantly different (range 0.0001–0.05 *P* level). The comparison of the results with antioxidant capacity and oxidative damage biomarkers concentrations showed that all changes indicating increased oxidative stress are directly related to ROS production increase. Therefore, the adopted method may be an automated technique for a lot of routine in clinical trials.

## 1. Introduction

Cells are exposed to a large variety of Reactive Oxygen Species (ROS) from both exogenous (i.e., pollutants, radiation) and endogenous sources: the latter are the majority, while mitochondria are the main source of their formation. At an appropriate concentration, ROS are known to act as important signaling molecules, essential to cell viability, playing various regulatory roles inside them [1]. Nevertheless enhanced levels of ROS overwhelming the cellular antioxidant defense system, phenomenon called oxidative stress, are implicated in the damage of cellular lipids, proteins, and DNA [1–3], increase cellular swelling, and decrease cell membrane fluidity.

High levels of ROS result in several degenerative processes and are involved in the etiopathogenesis of several pathological states, for example, cardiovascular (i.e., ischemia-reperfusion), neurodegenerative (i.e., Parkinson, Alzheimer, Amyotrophic Lateral Sclerosis, Multiple Sclerosis) [4], and inflammatory diseases, cancer [5], and so forth [6].

ROS also play a key role in a physiological process like aging. Indeed many reviews have been published regarding oxidative stress and theory of aging postulating a role of ROS in the pathogenesis of aging-related diseases [7, 8].

Direct measurements of ROS production are very difficult due to their high reactivity and low steady-state concentration [9]. Thus, oxidative stress assessment is usually performed by indirect methods measuring ROS-induced damage on proteins, membrane lipids, and DNA, the most vulnerable biological targets for oxidative stress [10, 11]. Thousands of articles have evaluated oxidative damage by measuring the increase of protein oxidation, by assessing Protein Carbonyls (PC), as well as lipid peroxidation, by determining among others Thiobarbituric Acid Reactive Substances (TBARS), in human blood (primarily in plasma, but also in serum or blood cells samples). These biomarkers were altogether found implicated in several pathologies. However enzymatic assays can be viewed as “a posteriori” methods with respect to Electron Paramagnetic Resonance (EPR), the unique technique capable of providing direct detection of the “instantaneous” presence of free radical species in a sample [12]. Nevertheless, despite the great potential of EPR technique, up to now, its use has been far from dominant: spare works report about free radicals measurement both in healthy subjects and in patients affected by various diseases, moreover carried out simply as qualitative methods [13]. It's unlikely that clinical EPR will ever become as extensively and widely utilized as clinical Nuclear Magnetic Resonance, but for everyone it is important to know the types of measurements that can be performed and their potential clinical applications. In fact, EPR may be able to provide useful information in a way that offers significant advantages over other approaches. EPR measurements are possible in vivo using spin probe or trapping, soluble paramagnetic materials or labels, returning the possibility of direct observation of ROS in cells and tissues. Although the experimental application in animals has been very successful [14], the technique has not yet been translated to clinical application. The vast majority of relevant human studies have measured redox status by using plasma or serum. This choice was probably adopted after considering that plasma better reflects tissue redox status [11] together with the ease of plasma collecting procedure. However, by this latter choice, potential artifacts generated by in vitro chemistry during sample preparation cannot be excluded. Moreover, classic tests have mainly quantified ROS levels in human plasma but not those associated with circulating cells, so excluding oxidant-scavenging abilities of these latter. Indeed red blood cells (RBC) may exert both antioxidant and prooxidant activity because of the high-iron concentration depending on the environmental milieu [15].

The goal of the present study was to evaluate the usefulness of the EPR technique for the study of ROS production in biology and medicine by applying an innovative microinvasive analytical approach [16] as suitable to be routinely applied under various pathophysiological states. The methodological study was carried out in two steps.

Firstly (step I) we aimed at demonstrating the reliability of our choice to measure ROS production rate in human capillary blood rather than in plasma as it is usually made. To this purpose, the relationship between EPR data collected on capillary versus venous blood and those obtained from its major components (i.e., plasma and erythrocytes) was assessed.

Once that the suitability of a widespread use of the aforementioned choice was confirmed, the potential application of the method as a routine microinvasive analytical tool to assess “ex vivo” oxidative stress in several physiological and pathological conditions was investigated (step II). To this aim, ROS absolute concentration level was measured in human capillary blood from the most various subjects' categories: young versus old and healthy versus diseased people at rest condition. A comparison of the results with antioxidant capacity and oxidative stress biomarkers concentrations determined in the same experiments was attempted too.

## 2. Materials and Methods

### 2.1. Subjects

*Step I*. ROS production in human blood and its components was assessed in one hundred healthy middle-aged sedentary (MS) subjects (34 males (M) and 66 females (F)).

*Step II*. ROS production was assessed in capillary blood of seven different subjects' categories: healthy subjects including (1) young elite athletes (YA, *n* = 18; male hockey players from the Varese hockey team), (2) young sedentary (YS, *n* = 32; M), (3) middle-aged sedentary (see step I), and (4) old sedentary subjects (OS, *n* = 68; 34 M and 34 F) and subjects affected by (5) sarcopenia (SAR, *n* = 25; 13 M and 12 F; primary sarcopenia: diagnosis in accordance with European consensus) [17], (6) Mild Cognitive Impairment (MCI *n* = 19; 10 M and 9 F; diagnosis in accordance with international guidelines) [18], and (7) sporadic Amyotrophic Lateral Sclerosis (sALS *n* = 22; 14 M and 8 F; diagnosis in accordance with international guidelines); ALS Functional Rating Scale Revised, score: 40.1 ± 3.7 (48: normal function; 0: severe disability) [19].

The age and anthropometric characteristics of the participants are reported in Table 1. In “Healthy” subjects, exclusion criteria concerning obesity, smoke, alcohol, and current use of medicines, special diet, minerals, vitamins, or other kind of supplementation as well as antioxidant supply in the time course of the study were applied. “Diseased” subjects showing evidence of target-organ or systemic damage were excluded, as well as, in the time course of the study, smoke, alcohol, special diets, minerals, vitamins or other kind of supplementation and/or antioxidant supply. Administration of antioxidants has been allowed only to sALS patients: in these subjects supplementation of vitamins (C, D, and E) and nutritional supplements such as coenzyme Q10 are currently recommended [20]. All sALS subjects were in pharmacological treatment with Riluzole.

All participants, after being informed of all risks, discomforts, and benefits involved in the study, signed a written informed consent. Procedures were in accordance with the Declaration of Helsinki, and institutional review board approval was received for this study.

### 2.2. Blood Samples

Approximately 3 mL of venous human blood was drawn from an antecubital vein, with subjects lying on a bed. The samples were collected in heparinized Vacutainer tubes (Vacutainer, Becton Dickinson, USA). Plasma was separated by centrifuge (5702R, Eppendorf, Germany) at 1000 ×g for 10 min at 4°C. After removal of plasma and discarding the buffy coat, aliquots of RBC were collected. 50 *μ*L of venous blood and of each prevalent blood component (i.e., plasma and RBC), immediately after preparation, was transferred to EPR determinations. 50 *μ*L capillary blood was taken from the fingertip and collected in heparinized capillary tubes (Cholestech LDX, Germany).

Plasma samples were stored in multiple aliquots at −80°C until assayed for oxidative stress biomarkers determination. Samples were thawed only once before analysis, performed within two weeks from collection.

### 2.3. EPR Measurements

An X-band EPR instrument (E-Scan—Bruker BioSpin, GmbH, MA USA) was adopted for determinations. The instrument allows us to deal with very low-concentration amounts of paramagnetic species in small (50 *μ*L) samples. As is well known, ROS half-life is too short if compared to the EPR time scale so that they result in being EPR-invisible but become EPR detectable once “trapped” and transformed in a more stable radical species. Among spin trapping or spin probe molecules, suitable for biological utilization, CMH (1-hydroxy-3-methoxycarbonyl-2,2,5,5-tetramethylpyrrolidine) probe was adopted.

**Figure 1:**
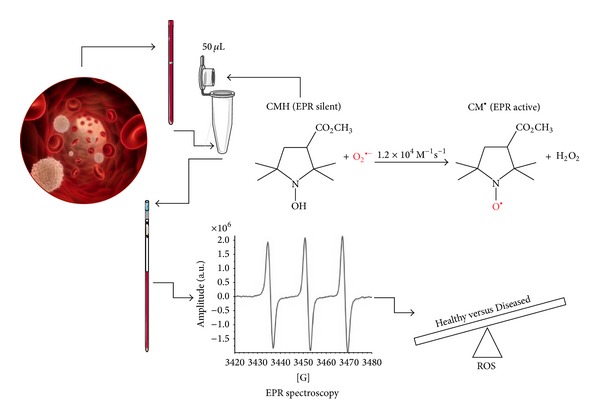
EPR sample preparation and acquisition protocol. CMH Spin Probe (50 *μ*L) is added in equal amount (1 : 1) to the collected capillary blood. The solution is immediately put in a glass EPR tube. From the generated radical compound, in the time course of the reaction, ten EPR spectra are collected in about 6 minutes, one of which is shown. The signal amplitude (a.u.) is proportional to the number of paramagnetic spin formed at the acquisition time. The calculated rate production values, converted in absolute levels (*μ*mol*·*min^−1^) by using CP ^∙^ radical as external standard, allowed us to significantly discriminate the analyzed subjects' groups.

For each recruited subject, ROS production rate was determined at rest by means of a recently developed EPR method [16] analyzing 50 *μ*L samples immediately treated with CMH solution (1 : 1). 50 *μ*L of the obtained solution was put in a glass EPR capillary tube (Noxygen Science Transfer & Diagnostics, Germany) that was placed inside the cavity of the E-scan spectrometer for data acquisition (Figure 1).

Acquisition parameters were microwave frequency 9.652 GHz; modulation frequency 86 kHz; modulation amplitude 2.28 G; sweep width 60 G, microwave power 21.90 mW, number of scans 10; and receiver gain 3.17·10^1^. Sample temperature was firstly stabilized and then kept at 37°C by the Temperature & Gas Controller “Bio III” unit, interfaced to the spectrometer. Spectra were recorded and analyzed by using Win EPR software (2.11 version) standardly supplied by Bruker.

EPR measurements allowed us to attain a relative quantitative determination of ROS production rate in samples. All data were, in turn, converted in absolute concentration levels (*μ*mol*·*min^−1^) by adopting CP ^∙^ (3-Carboxy-2,2,5,5-tetramethyl-1-pyrrolidinyloxy) stable radical as external reference.

### 2.4. Enzymatic Assays

Because of difficulty in measuring each antioxidant component separately and interactions among antioxidants, the adopted here method assesses the total antioxidant capacity (TAC) of plasma. Protein Carbonyls and Thiobarbituric Acid Reactive Substances were also determined and used as protein damage and lipid peroxidation standard indices, respectively.

#### 2.4.1. Total Antioxidant Capacity (TAC)

The 6-hydroxy-2,5,7,8-tetramethylchroman-2-carboxylic acid (Trolox-) equivalent antioxidant capacity assay, a widely used kit-based commercial method, was used. Briefly, 10 *μ*L of plasma is added in duplicate to 10 *μ*L of metmyoglobin and 150 *μ*L of the chromogen solution then; reactions are initiated by the addition of 40 *μ*L of hydrogen peroxide, as indicated by the manufacturer (Cayman Chemical, USA). Reaction mixtures are incubated for 3 min at room temperature and then read measuring the absorbance signal at 750 nm by an Infinite M200 microplate reader spectrophotometer (Tecan, Austria). A linear calibration curve is computed from pure Trolox-containing reactions.

#### 2.4.2. Protein Carbonyls (PC)

The accumulation of oxidized proteins was measured by content of reactive carbonyls. A Protein Carbonyl assay kit (Cayman Chemical, USA) was used to colorimetrically evaluate oxidized proteins. The samples were read at 370 nm, by a microplate reader spectrophotometer (Infinite M200, Tecan, Austria), as described in detail by the manufacturer. Oxidized proteins values obtained were normalized to the total protein concentration in the final pellet (absorbance reading at 280 nm), in order to consider protein loss during the washing steps, as suggested in the kit's user manual.

#### 2.4.3. Thiobarbituric Acid Reactive Substances (TBARS)

We used TBARS assay kit (Cayman Chemical, USA) which allows a rapid photometric detection of the Thiobarbituric Acid Malondialdehyde (TBAMDA) adduct at 532 nm. Samples were read by a microplate reader spectrophotometer (Infinite M200, Tecan, Austria). A linear calibration curve was computed from pure MDA-containing reactions.

All samples were determined in duplicate and the interassay coefficient of variation was in the range indicated by the manufacturer.

### 2.5. Statistical Analysis

Numerical values are reported as mean ± standard deviation (SD). Data were analyzed using parametric statistics following mathematical confirmation of a normal distribution using Shapiro-Wilks *W* test.

To estimate the significance of ROS production rate in blood (capillary versus venous, plasma, or erythrocytes) one-way ANOVA with Bonferroni post hoc test was applied. Oxidative stress (ROS, TBARS, PC, and TAC) experimental data were compared using repeated ANOVA measurements with Holm-Sidak's multiple comparisons test. The relationship between selected variables was assessed using Pearson Correlation coefficients. All statistical analyses were performed by using GraphPad Prism package (GraphPad Prism 6, Software Inc. San Diego, CA). A *P* value < 0.05 was considered statistically significant.

## 3. Results

### 3.1. Step I: ROS Production Rate in Blood Components

**Figure 2:**
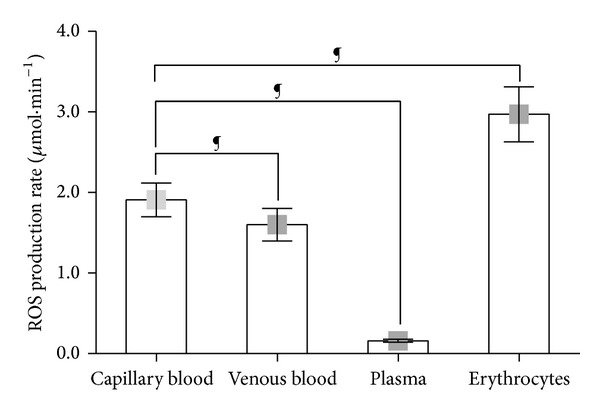
Histogram plot (mean ± SD) of the absolute ROS production rate (*μ*mol*·*min^−1^) obtained from capillary blood; venous blood; plasma; and erythrocytes in the same resting healthy subjects. ^¶^ *P* < 0.0001 significant differences.

ROS production rate in human blood and components is shown in Figure 2. Different ROS concentrations were obtained by recording data, on the same resting subject, from the different blood collections (i.e., capillary and venous) and fractions (i.e., plasma and RBC). In capillary blood ROS production level (*μ*mol*·*min^−1^) was found significantly greater than in venous (1.91 ± 0.20 versus 1.63 ± 0.29). The greatest level was found in samples containing only erythrocytes (2.96 ± 0.30). On the contrary significantly lower levels (0.16 ± 0.02) were found in plasma samples. Significant (*P* < 0.0001) differences between venous blood versus erythrocytes, venous versus plasma, and plasma versus erythrocytes were found. The results are not shown in Figure 2 in order to put in greater relevance the difference between capillary blood and the other samples.

**Figure 3:**
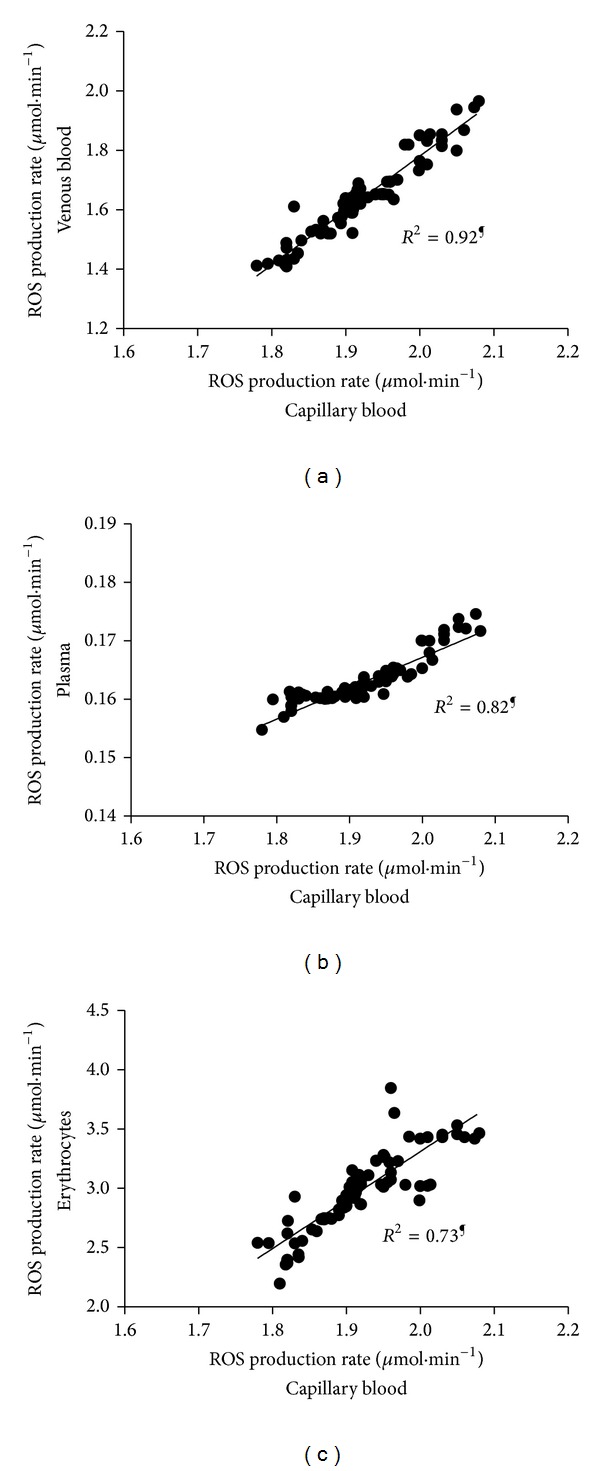
Multipanel plots of ROS production rate levels (*μ*mol*·*min^−1^) measured in capillary blood versus (a) venous blood; (b) plasma; and (c) erythrocytes (full symbols). The linear regression fit (solid line) is also shown and so is the correlation coefficient (*R* ^2^) reported in each panel. A significant linear relationship (^¶^ *P* < 0.0001) in the ROS production rate between capillary blood and the other blood components was estimated.

The multipanel plots reported in Figure 3 show a significant linear relationship between the ROS production rate found in capillary blood and (a) venous blood (*R* ^2^ = 0.92); (b) plasma (*R* ^2^ = 0.82); and (c) erythrocytes (*R* ^2^ = 0.73).

### 3.2. Step II: ROS Production Rate and Oxidative Damage: Resting Data

**Figure 4:**
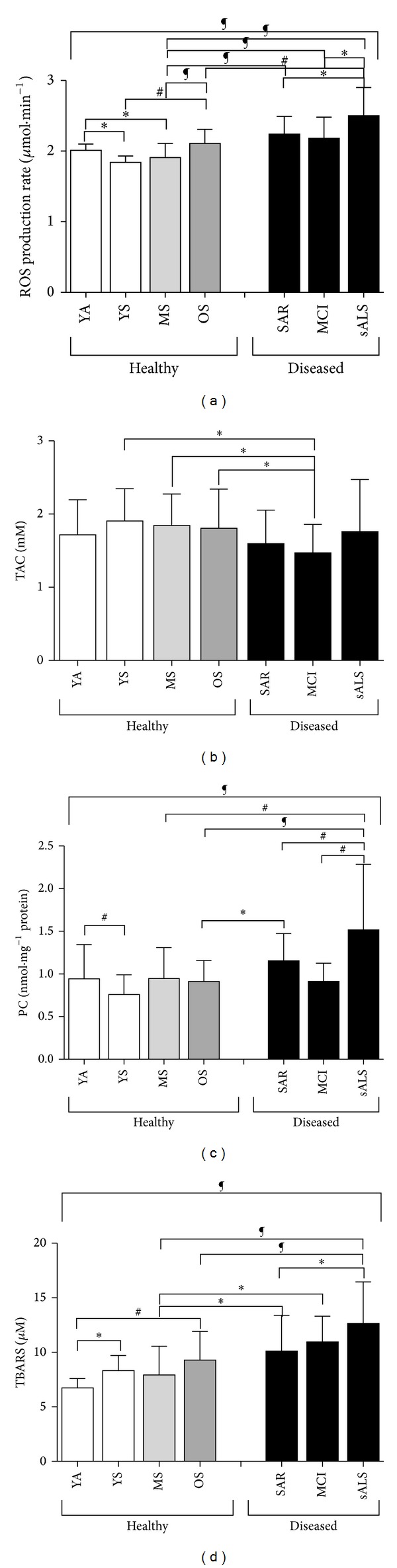
Histogram plot (mean ± SD) of (a) ROS production rate obtained from capillary blood samples and plasma concentration levels of (b) TAC, (c) PC, and (d) TBARS, collected at baseline (rest condition) in healthy (YA, YS, MS, and OS) and pathological subjects (SAR, MCI, and sALS). *P < 0.05, ^#^ *P* < 0.01, ^§^ *P* < 0.001, and ^¶^ *P* < 0.0001 significant difference.

ROS production rate in capillary blood (Figure 4(a)), TAC (Figure 4(b)) and oxidative stress biomarkers concentrations, PC (Figure 4(c)) and TBARS (Figure 4(d)) in plasma, assessed in healthy and pathological subjects are displayed. The statistically significant differences, calculated from the collected data between different groups, that is, healthy subjects (YA, YS, MS, and OS) and diseased subjects (SAR, MCI, and sALS), are hereinafter reported. In particular (Figure 4(a)), a significantly different ROS production level (*μ*mol*·*min^−1^) between healthy and diseased groups was found. In greater detail, significant differences were calculated as follows:

- in the healthy group: YA (2.01 ± 0.09) versus YS (1.84 ± 0.09) and MS (1.91 ± 0.21); OS (2.12 ± 0.29) versus YS (1.84 ± 0.09), and MS (1.91 ± 0.21);
- between the healthy and the diseased groups: MS (1.91 ± 0.21) versus: SAR (2.24 ± 0.26), MCI (2.18 ± 0.31) and, sALS (2.49 ± 0.43); OS versus sALS (2.12 ± 0.29 versus 2.49 ± 0.43);
- in the diseased groups: SAR versus sALS (2.24 ± 0.26 versus 2.49 ± 0.43) and MCI versus sALS (2.18 ± 0.31 versus 2.49 ± 0.43).

In TAC (mM) (Figure 4(b)) significant differences between the healthy and the diseased groups are as follows: MCI (1.47 ± 0.38) versus YS (1.91 ± 0.44), MS (1.84 ± 0.43) and OS (1.81 ± 0.53) are shown.

Significant difference in PC (nmol*·*mg^−1^ protein) concentration between healthy and diseased groups shows up (Figure 4(c)). In greater detail significant differences were calculated as follows:

- in the healthy groups: YA versus YS (0.94 ± 0.40 versus 0.76 ± 0.23);
- between the healthy and the pathological groups: MS versus sALS (0.94 ± 0.36 versus 1.52 ± 0.77), OS (0.91 ± 0.24) versus SAR (1.15 ± 0.32), and sALS (1.52 ± 0.77);
- in the diseased groups: sALS (1.52 ± 0.77) versus SAR (1.15 ± 0.32) and MCI (0.91 ± 0.21).

Concerning TBARS concentration (*μ*M) (Figure 4(d)) significant differences between healthy and diseased groups resulted and, in detail, significant differences were calculated as follows:

- in the healthy group: YA (6.74 ± 0.84) versus YS (8.33 ± 1.38) and OS (9.29 ± 2.61);
- between the healthy and the diseased groups: MS (7.92 ± 2.64) versus SAR (10.12 ± 3.28), MCI (10.96 ± 2.34), and sALS (12.66 ± 3.79); OS versus sALS (9.29 ± 2.61 versus 12.66 ± 3.79);
- in the diseased groups: SAR versus sALS (10.12 ± 3.28 versus 12.66 ± 3.79).

Finally, by considering YS and YA groups taken all together versus single components of the disease group, differences were found as regard as: (i) ROS production rate levels: (SAR *P* < 0.001; MCI *P* < 0.001; sALS *P* < 0.0001); (ii) PC: (SAR *P* < 0.001; sALS *P* < 0.0001), and (iii) TBARS: (SAR *P* < 0.001; MCI *P* < 0.001; sALS *P* < 0.0001) (data not shown).

**Figure 5:**
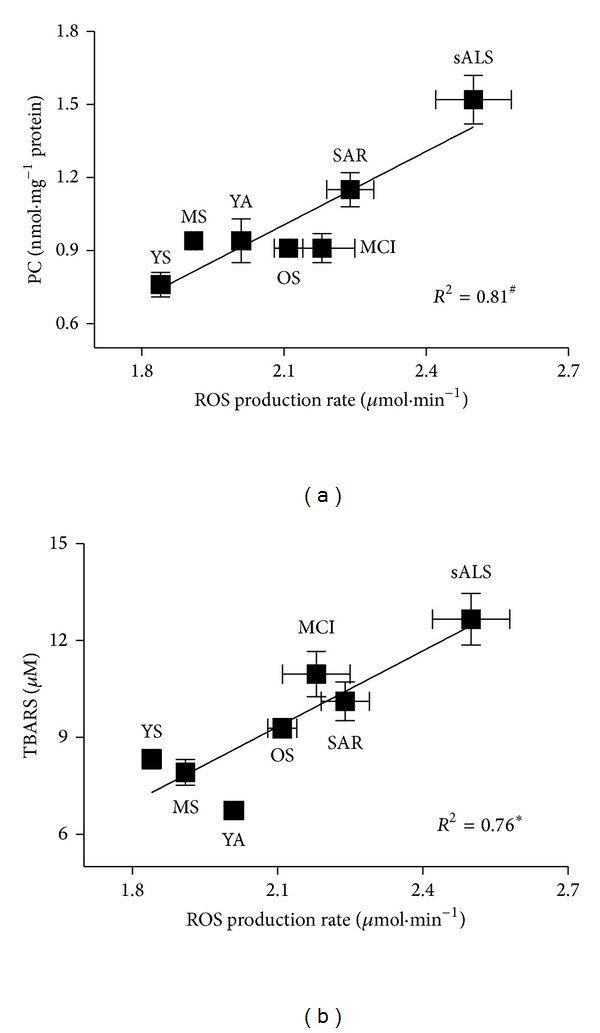
Panel plots (for appropriate graphical displays data are reported as mean ± standard error of means—SEM) of ROS production rate levels (*μ*mol*·*min^−1^) measured in capillary blood versus (a) PC and (b) TBARS concentrations. The linear regression fit (solid line) is also shown and so is the correlation coefficient (*R* ^2^) reported in each panel. A significant linear relationship in the ROS production rate between PC (*P* < 0.01) and TBARS (*P* < 0.05) values was estimated.

Figure 5 shows the plot of the correlation between plasma PC (Figure 5(a)) and TBARS (Figure 5(b)) and ROS production values of capillary blood at rest in the examined groups of subjects. A positive linear relationship was found at rest between ROS production and plasma PC (*R* ^2^ = 0.81) and TBARS (*R* ^2^ = 0.76) concentrations.

## 4. Discussion

The most significant challenges that investigators face in their attempt to directly assess oxidant levels in vivo are both the low concentration of ROS present and the extremely transient nature of these species. The role of free radicals, generated in the biological milieu and propagated through a cascade of reactions, has been well recognized in the pathogenesis of many diseases, including a variety of inflammatory and degenerative pathologies. Much progress has been made in ROS direct detection, characterization, and quantification. Indeed Electron Paramagnetic Resonance Spectroscopy detects free radicals and paramagnetic molecules and plays a major role in the assessment of most of the oxidants characterized by very short half-life (nanoseconds to microseconds) usually by using stabilizing molecules called spin-traps/probes. The magnetic field-based EPR detection enables nondestructive (in vitro) and noninvasive (in vivo) measurements of biological samples. Therefore EPR spectroscopy, coupled with the use of paramagnetic probes, has become a potential technique for accurate and precise determination of ROS concentrations in a variety of biological samples. It was shown [21, 22] that cyclic hydroxylamines as CMH can be used to assay formation of reactive oxidant species resulting from differently generated oxidative stress, detecting a 10-fold lower superoxide radical level than other spin-traps, thereby providing superior sensitivity for quantification of superoxide radical in vitro. Moreover, it was found that the use of EPR detection of CMH to measure the formation of reactive oxidant species in blood is a very sensitive and reproducible method. The superior sensitivity of CMH for superoxide detection provides a relevant method to assay superoxide and other reactive oxidant species able to induce oxidative stress under different experimental conditions. Among all tested hydroxylamines, CMH showed very high cellular accumulation and the highest rate of intracellular nitroxide formation. In addition the high cell permeability makes it suitable to detect both extra- and intracellular ROS production.

Till now, due to technical difficulties in directly assessing oxidant levels in vivo, investigators have generally had to rely on indirect oxidative stress measurements techniques. In particular, redox system evaluation, so that the assessments of oxidative damage biomarkers is widely adopted as standard indirect method for the determination of ROS production.

The purpose of this study was to evaluate the usefulness of the EPR technique for the study of ROS in biology and medicine by applying an innovative analytical approach [16].

Data resulting from the parallel determination of ROS production reported in Figure 2 well demonstrate the crucial role played by the choice of the sample examined on the results obtained, suggesting that great care must be taken in setting parameters and experimental procedure. Indeed, as can be easily observed, different results were collected in the same resting subject, from peripheral capillary blood, with respect to venous samples. At the same time red blood cells rather than plasma sample analysis gave different values too. Indeed also the other blood corpuscular components contribute to ROS production [23, 24]. Nevertheless their preparation is a complex and time-consuming procedure: in particular sample manipulation might affect results giving rise to artifacts. Moreover, in the examined experimental conditions, they constitute only a minimal part of total blood amount. For all these reasons an analysis of corpuscular components was not carried out here nor was it considered peculiar to this study. As expected significantly lower ROS production rate levels were found from plasma samples due to the absence of RBC, the main ROS producing elements in blood. Capillary blood data were found greater than those collected on venous blood, owing to the different oxygen concentration in the sample (PO_2_ = 100 mmHg versus 75 mmHg resp.) showing that EPR data are extremely sensitive to PO_2_ so that measurement have to be taken with great accuracy. At the same time, erythrocytes' samples were characterized by the greatest levels due to the increased number of red cells present in the sample. Nevertheless as shows from Figure 3 a linear relationship with good correlation factor was anyway found between capillary blood versus the other examined samples. The high correlation (*R* ^2^ = 0.92) with venous blood (Figure 3(a)) is expected due to the constant difference between the PO_2_ levels at rest condition. The good correlation (*R* ^2^ = 0.73) with erythrocytes (Figure 3(c)), as expected, reveals the sensitivity of EPR technique to the number of spin involved in the measurement, at the same time suggesting that care must be taken in assuring that the results are not influenced by the hematocrit value. As a matter of fact, the vast majority of the relevant human studies have measured the redox status using plasma or serum. This choice was adopted after considering that plasma better reflects tissue redox status together with the ease of plasma collecting procedure. Indeed a good correlation (*R* ^2^ = 0.82) between ROS production rates in capillary blood and in plasma samples (Figure 3(b)) assessed in parallel determinations was found. All these considerations well validate our choice to make determinations of Step II anyway using capillary blood aiming to reduce the invasiveness of the method and hence increase its clinical potential in pathological enhancement.

The herein adopted method allows reliable, rapid, and noninvasive measurement of the instantaneous ROS concentration levels directly in human peripheral blood. Due to its simplicity coupled with the high sensitivity and specificity of EPR technique, its use has successfully been validated both at rest and after interventions able to alter the redox status of a living system [16].

Main aim of the second phase of the study (step II) was to test whether the method was suitable to detect significant differences in heterogeneous categories of subjects, demonstrating its sensibility even under different both physiological (i.e., aging) and pathological (sarcopenia, MCI, and sALS) experimental conditions. The measurements of blood oxidative stress markers, a currently common practice in biomedical research, as indirect index of ROS production in pathophysiological conditions was examined too and correlation with EPR data was attempted.

Many reviews have been published regarding the oxidative stress theory of aging [1, 25–27]. Substantially higher levels of lipid peroxidation products have been observed in aged compared with young subjects [28]. In our study the observed ROS overproduction (Figure 4(a)), related to the aging process, caused oxidative damage as attested by increases in lipid peroxidation (Figure 4(d)) suggesting that the data follow a trend where redox imbalance leads to an activation of redox-sensitive transcription factors together with subsequent generation of numerous proinflammatory mediators. A common phenomenon in aging-related pathologies is the discovery of ROS as a potential unifying mechanism contributing to many of such diseases [29, 30].

In this study we focused on three types of pathological status in the genesis of which oxidative stress is hypothesized to be implicated: sarcopenia, Mild Cognitive Impairment, and Amyotrophic Lateral Sclerosis. The aberrant oxidative damage observed is likely to contribute to an imbalance that may presage the onset of age-related loss of skeletal muscle mass [31], cognitive impairment and neurodegenerative process respectively.

Oxidative damage is highly relevant in neuronal tissue since this latter is a postmitotic high oxygen consuming tissue characterized by poor concentration of antioxidants but rich in polyunsaturated fatty acids, biomacromolecules most susceptible to oxidation. In addition, the tissue is enriched with iron, which accumulates in brain as a function of age like a potentially potent catalyst for oxidative species formation. Indeed there is evidence that oxidative stress-mediated protein aggregation may be the primary cause of the neuronal death in several forms of aging-related neurodegenerative diseases [32].

Regardless of etiology, there is irrefutable evidence for some components of oxidative stress in all of these neurodegenerative diseases, even if the unresolved question is whether oxidative stress is a consequence of degenerative processes initiated by some other factor.

As for sarcopenia, combined effects of aging and lifelong inactivity, related to ROS overproduction generating oxidative damage, also play a role in regulating intracellular signal transduction pathways, directly or indirectly involved in skeletal muscle atrophy and alterations of muscle contractility.

It is important to underline that in all the examined pathological conditions here, even if different in both phenotypic manifestations and disease targets, our measurements reported common higher ROS production levels and corresponding higher oxidative damage biomarker concentrations (Figures 5(a) and 5(b)). Anyway the positive correlation between ROS production rate levels and the corresponding plasma TBARS and PC concentrations confirms, in healthy and diseased groups, our previous observations made in a homogeneous experimental group (young athletes) [16]. Although almost all oxidative stress biomarkers have been criticized for their reliability (including those used in the present study), it is apparent that, at rest, all changes indicating increased oxidative stress are directly related to ROS production (Figure 5).

## 5. Conclusion

In conclusion, EPR technique was demonstrated to offer unique advantages in the determination of free radicals and paramagnetic species in biological samples. The method adopted here is direct, definitive, noninvasive, sensitive, and quantitative so that it can be identified as indispensable tool in the study of oxidants and oxidative stress in free radical biology and medicine.

Our results suggest that an exaggerated production of ROS will lead to extensive oxidative damage and alterations in intracellular signal transduction, which subsequently might contribute to cellular and organ dysfunction and age-related reductions in stress tolerance. Indeed, in all experimental conditions examined, it is apparent that, at rest, all changes indicating increased oxidative stress are directly related to an increase in ROS production. Even if oxidative stress is likely not always the primary pathological insult, ROS production plays a contributory role in the disease process. Therefore the ROS production rate determination might be a hotspot on disease progression, an efficacy drug screening, and even a therapeutic target.

In conclusion the primary goal of demonstrating the adopted method here like an automated technique for a lot of routine in clinical trials has been successfully achieved. Aware that it's hard to consider EPR a diagnostic tool as extensively and widely utilized as clinical Nuclear Magnetic Resonance, we think EPR may be able to provide useful information in a way that offers significant advantages over other approaches.

## Figures and Tables

**Table 1:** 

Subjects' features (Caucasian-Italian people)							
	Healthy				Diseased		
	YA	YS	MS	OS	SAR	MCI	sALS
	(*n* = 18)	(*n* = 32)	(*n* = 100)	(*n* = 68)	(*n* = 25)	(*n* = 19)	(*n* = 22)
Age (yr)	19.7 ± 1.1	23.0 ± 1.0	47 ± 5.2	72.1 ± 4.5	75.0 ± 5.1	74.3 ± 5.4	56.9 ± 11.9
Weight (kg)	77.6 ± 6.9	78.0 ± 4.8	71.3 ± 8.1	68.8 ± 12.0	79.9 ± 15.6	67.2 ± 12.8	67.6 ± 11.8
Height (m)	1.78 ± 0.04	1.79 ± 0.05	1.69 ± 0.09	1.68 ± 0.09	1.73 ± 0.05	1.64 ± 0.08	1.69 ± 0.08
BMI (kg*·*m^−2^)	24.6 ± 2.0	23.4 ± 1.8	25.0 ± 3.2	24.1 ± 3.1	27.7 ± 4.2	24.9 ± 3.0	23.58 ± 3.0
